# Crystal structure of 1*H*-imidazol-3-ium 2-(1,3-dioxoisoindolin-2-yl)acetate

**DOI:** 10.1107/S1600536814017619

**Published:** 2014-08-06

**Authors:** Shaaban K. Mohamed, Mehmet Akkurt, Herman Potgieter, Muizz Ali

**Affiliations:** a Chemistry and Environmental Division, Manchester Metropolitan University, Manchester M1 5GD, England; b Chemistry Department, Faculty of Science, Minia University, 61519 El-Minia, Egypt; c Department of Physics, Faculty of Sciences, Erciyes University, 38039 Kayseri, Turkey; d Analytical Development Division, Manchester Metropolitan University, Manchester M1 5GD, England

**Keywords:** crystal structure, 1*H*-imidazol-3-ium salt, 2-(1,3-dioxoisoindolin-2-yl)acetate salt, hydrogen bonding, π–π stacking inter­actions, co-crystallization, pharmaceuticals

## Abstract

The title salt, C_3_H_5_N_2_^+^·C_10_H_6_NO_4_^−^, was obtained during a study of the co-crystallization of *N*′-[bis­(1*H*-imidazol-1-yl)methyl­ene]isonicotinohydrazide with (1,3-dioxoisoindolin-2-yl)acetic acid under aqueous conditions. The 1,3-dioxoisoindolinyl ring system of the anion is essentially planar [maximum deviation = 0.023 (2) Å]. In the crystal, cations and anions are linked *via* classical N—H⋯O hydrogen bonds and weak C—H⋯O hydrogen bonds, forming a three-dimensional network. Weak C—H⋯π inter­actions and π–π stacking inter­actions [centroid–centroid distances = 3.4728 (13) and 3.7339 (13) Å] also occur in the crystal.

## Related literature   

For the use of co-crystals in drug design, see: Babu & Nangia (2011 ▶); Sekhon (2013 ▶); Frantz (2006 ▶); Pan *et al.* (2008 ▶); Vermeire *et al.* (2001 ▶).
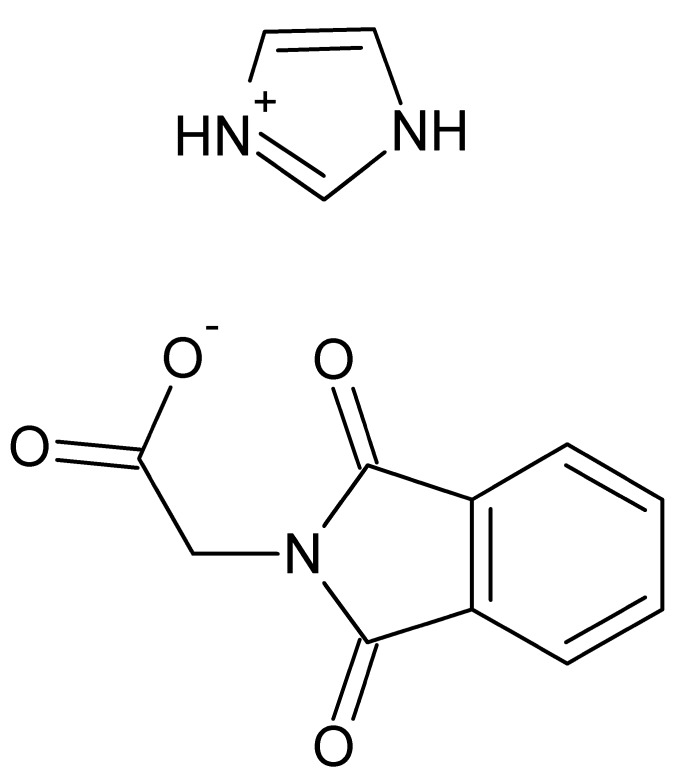


## Experimental   

### Crystal data  

C_3_H_5_N_2_^+^·C_10_H_6_NO_4_^−^
*M* *_r_* = 273.25
Monoclinic, 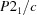

*a* = 9.8750 (7) Å
*b* = 18.0543 (15) Å
*c* = 7.0942 (5) Å
β = 100.955 (7)°
*V* = 1241.75 (16) Å^3^
*Z* = 4
Mo *K*α radiation
μ = 0.11 mm^−1^
*T* = 150 K
0.09 × 0.02 × 0.02 mm

### Data collection  

Agilent SuperNova, Single source at offset, Eos diffractometer
Absorption correction: multi-scan (*CrysAlis PRO*; Agilent, 2013 ▶) *T*_min_ = 0.859, *T*_max_ = 1.000
4781 measured reflections
2756 independent reflections
1993 reflections with *I* > 2σ(*I*)
*R*_int_ = 0.028

### Refinement  

*R*[*F* ^2^ > 2σ(*F* ^2^)] = 0.053
*wR*(*F* ^2^) = 0.118
*S* = 1.06
2756 reflections
189 parameters
H atoms treated by a mixture of independent and constrained refinement
Δρ_max_ = 0.26 e Å^−3^
Δρ_min_ = −0.26 e Å^−3^

### 

Data collection: *CrysAlis PRO* (Agilent, 2013 ▶); cell refinement: *CrysAlis PRO*; data reduction: *CrysAlis PRO*; program(s) used to solve structure: *SHELXS2013* (Sheldrick, 2008 ▶); program(s) used to refine structure: *SHELXL2013* (Sheldrick, 2008 ▶); molecular graphics: *ORTEP-3 for Windows* (Farrugia, 2012 ▶) and *PLATON* (Spek, 2009 ▶); software used to prepare material for publication: *PLATON* (Spek, 2009 ▶).

## Supplementary Material

Crystal structure: contains datablock(s) global, I. DOI: 10.1107/S1600536814017619/xu5807sup1.cif

Structure factors: contains datablock(s) I. DOI: 10.1107/S1600536814017619/xu5807Isup2.hkl

Supporting information file. DOI: 10.1107/S1600536814017619/xu5807Isup3.cml

**Fig. 1.**
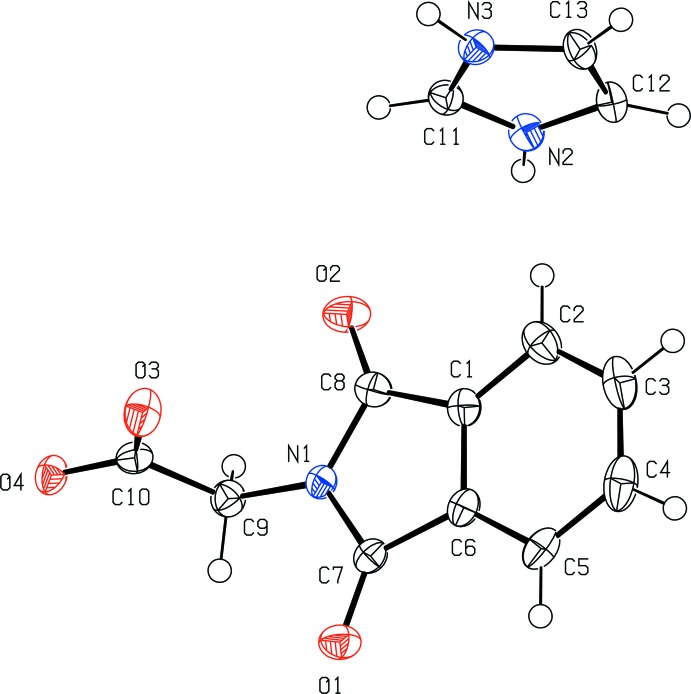
Perspective view of the title compound (I). Displacement ellipsoids are drawn at the 50% probability level.

**Fig. 2.**
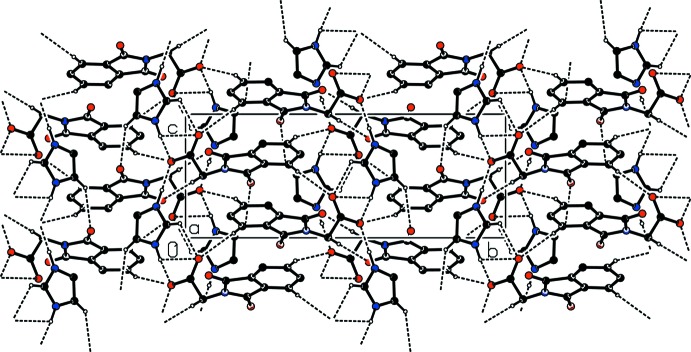
Packing viewed down the a axis showing the intermolecular interactions as dotted lines.

. DOI: 10.1107/S1600536814017619/xu5807fig1.tif
Perspective view of the title compound (I). Displacement ellipsoids are drawn at the 50% probability level.

a . DOI: 10.1107/S1600536814017619/xu5807fig2.tif
Packing viewed down the *a* axis showing the inter­molecular inter­actions as dotted lines.

CCDC reference: 1017262

Additional supporting information:  crystallographic information; 3D view; checkCIF report

## Figures and Tables

**Table 1:** Hydrogen-bond geometry (Å, °) *Cg*4 is the centroid of the N2/N3/C11–C13 ring.

*D*—H⋯*A*	*D*—H	H⋯*A*	*D*⋯*A*	*D*—H⋯*A*
N2—H2*N*⋯O4^i^	0.99 (3)	1.69 (3)	2.6846 (19)	178 (4)
N3—H3*N*⋯O4^ii^	0.97 (2)	1.71 (2)	2.680 (2)	175 (2)
C3—H3⋯O4^iii^	0.95	2.45	3.321 (3)	153
C5—H5⋯O3^iv^	0.95	2.48	3.266 (3)	141
C9—H9*B*⋯O2^i^	0.99	2.41	3.397 (3)	172
C11—H11⋯O3^ii^	0.95	2.40	2.987 (3)	120
C13—H13⋯O1^v^	0.95	2.54	3.352 (2)	143
C2—H2⋯*Cg*4	0.95	2.87	3.805 (2)	166

Symmetry codes: (i) 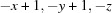; (ii) 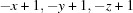; (iii) 
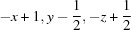
; (iv) 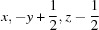; (v) 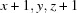.

## supplementary crystallographic information

### S1. Comment

The use of co-crystals in drug design see and delivery and as functional materials with potential applications as pharmaceuticals has recently attracted a significant amount of interest in the pharmaceutical industry (Babu & Nangia, 2011). Co-crystallization in particular is a reliable technique for the modification of the physical properties of a drug as it enables the control of physical properties of Active Pharmaceutical Ingredient (API) molecules such as dissolution, stability, solubility, bioavailability, hygroscopisity and compressibility without changing the chemical composition of the API (Sekhon, 2013). Multi-API co-crystals are also possible solid forms for the delivery of combination drugs that can be tested to overcome problems related with traditional combination drugs (Frantz, 2006). Another benefit of multi-API co-crystal is the ability to reduce the number of pills being taken by a patient due to the improvement of patients long-term medication compliance in long-term drug therapy, since fewer pills need to be taken (Pan *et al.*, 2008; Vermeire *et al.*, 2001). The title compound was obtained during our study on co-crystallization reaction of *N*'-(di-1*H*-imidazol-1-ylmethylene)isonicotinohydrazide with (1,3-dioxoisoindolin-2-yl)acetic acid under aqouse condition.

Fig. 1 shows one 1*H*-imidazol-3-ium cation and one (1,3-dioxoisoindolin-2-yl)acetate anion in the asymmetric unit of the title compound (I).

The five-membered ring (N2/N3/C11—C13) of the 1*H*-imidazol-3-ium cation is essentially planar [maximum deviation = 0.003 (2) Å for C12]. The nine-membered ring system (N1/C1–C8) of the (1,3-dioxoisoindolin-2-yl)acetate anion is also essentially planar [maximum deviation = -0.023 (2) Å for C8].

In the crystal structure, the anions and cations of (I) are linked *via* N—H···O and C—H···O hydrogen bonds (Table 1, Fig. 2), forming three dimensional network. Further C—H···π interactions (Table 1) and face-to-face π-π stacking interactions [*Cg*1···*Cg*2 (*x*, 1/2 - *y*, -1/2 + *z*) = 3.4728 (13) Å, Cg2···Cg2 (x, 1/2-y, 1/2+z) = 3.7339 (13) Å, where *Cg*1 and *Cg*2 are the centroids of the N1/C1/C6–C8 and C1–C6 rings, respectively] presents in the three-dimensional framework.

### S2. Experimental

A mixture of 1 mmol (281 mg) of *N*'-(di-1*H*-imidazol-1-ylmethylene)isonicotinohydrazide and 1 mmol (205 mg) of (1,3-dioxoisoindolin-2-yl)acetic acid was stirred in 30 ml ethanol at room temperature. Few drops of glacial acetic acid as a catalyst was added to the reaction mixture and allowed to reflux at 351 K for 5 h. The reaction progress was monitored by TLC using a mixture of cyclohexane and ethyl acetate (1:1) as an eluent. On completion, the reaction mixture was poured on crushed ice (50 g). The resulting solid was filtered off, washed with cold ethanol dried under vacuum and recrystallized from ethanol to yield colourless blocks of the title compound (74% yield).

### S3. Refinement

H atoms attached to carbon were placed in calculated positions (C—H = 0.95 and 0.99 Å) and were included as riding contributions with isotropic displacement parameters 1.2 those of the attached atoms. H-atoms attached to nitrogen were placed in locations derived from a difference map and they were refined freely.

### Crystal data

**Table:**

C_3_H_5_N_2_^+^·C_10_H_6_NO_4_^−^	*F*(000) = 568
*M**_r_* = 273.25	*D*_x_ = 1.462 Mg m^−^^3^
Monoclinic, *P*2_1_/*c*	Mo *K*α radiation, λ = 0.7107 Å
Hall symbol: -P 2ybc	Cell parameters from 1373 reflections
*a* = 9.8750 (7) Å	θ = 4.0–27.4°
*b* = 18.0543 (15) Å	µ = 0.11 mm^−^^1^
*c* = 7.0942 (5) Å	*T* = 150 K
β = 100.955 (7)°	Block, colourless
*V* = 1241.75 (16) Å^3^	0.09 × 0.02 × 0.02 mm
*Z* = 4	

### Data collection

**Table:**

Agilent SuperNova, Single source at offset, Eos diffractometer	2756 independent reflections
Radiation source: SuperNova (Mo) X-ray Source	1993 reflections with *I* > 2σ(*I*)
Mirror monochromator	*R*_int_ = 0.028
Detector resolution: 8.0714 pixels mm^-1^	θ_max_ = 29.1°, θ_min_ = 3.1°
ω scans	*h* = −12→12
Absorption correction: multi-scan (*CrysAlis PRO*; Agilent, 2013)	*k* = −9→23
*T*_min_ = 0.859, *T*_max_ = 1.000	*l* = −9→5
4781 measured reflections	

### Refinement

**Table:**

Refinement on *F*^2^	0 restraints
Least-squares matrix: full	Hydrogen site location: inferred from neighbouring sites
*R*[*F*^2^ > 2σ(*F*^2^)] = 0.053	H atoms treated by a mixture of independent and constrained refinement
*wR*(*F*^2^) = 0.118	*w* = 1/[σ^2^(*F*_o_^2^) + (0.0327*P*)^2^ + 0.2765*P*] where *P* = (*F*_o_^2^ + 2*F*_c_^2^)/3
*S* = 1.06	(Δ/σ)_max_ < 0.001
2756 reflections	Δρ_max_ = 0.26 e Å^−^^3^
189 parameters	Δρ_min_ = −0.26 e Å^−^^3^

### Special details

**Table:**

Geometry. Bond distances, angles *etc*. have been calculated using the rounded fractional coordinates. All su's are estimated from the variances of the (full) variance-covariance matrix. The cell e.s.d.'s are taken into account in the estimation of distances, angles and torsion angles
Refinement. Refinement on *F*^2^ for ALL reflections except those flagged by the user for potential systematic errors. Weighted *R*-factors *wR* and all goodnesses of fit *S* are based on *F*^2^, conventional *R*-factors *R* are based on *F*, with *F* set to zero for negative *F*^2^. The observed criterion of *F*^2^ > σ(*F*^2^) is used only for calculating -*R*-factor-obs *etc*. and is not relevant to the choice of reflections for refinement. *R*-factors based on *F*^2^ are statistically about twice as large as those based on *F*, and *R*-factors based on ALL data will be even larger.

### Fractional atomic coordinates and isotropic or equivalent isotropic displacement parameters (Å^2^)

**Table:**

	*x*	*y*	*z*	*U*_iso_*/*U*_eq_	
O1	0.16235 (14)	0.29586 (8)	−0.05484 (19)	0.0277 (5)	
O2	0.54966 (15)	0.42729 (9)	0.1952 (2)	0.0316 (5)	
O3	0.24304 (16)	0.45939 (9)	0.33073 (19)	0.0310 (5)	
O4	0.14750 (14)	0.54438 (8)	0.11691 (18)	0.0236 (5)	
N1	0.33840 (16)	0.37710 (10)	0.0589 (2)	0.0202 (5)	
C1	0.5087 (2)	0.29370 (12)	0.1790 (3)	0.0215 (6)	
C2	0.6297 (2)	0.25882 (14)	0.2609 (3)	0.0292 (7)	
C3	0.6299 (2)	0.18176 (14)	0.2585 (3)	0.0344 (8)	
C4	0.5146 (3)	0.14112 (14)	0.1752 (3)	0.0343 (8)	
C5	0.3917 (2)	0.17722 (12)	0.0932 (3)	0.0265 (7)	
C6	0.3923 (2)	0.25360 (12)	0.0988 (3)	0.0207 (6)	
C7	0.2809 (2)	0.30730 (12)	0.0234 (3)	0.0199 (6)	
C8	0.4768 (2)	0.37384 (13)	0.1526 (3)	0.0221 (6)	
C9	0.2655 (2)	0.44563 (12)	0.0027 (3)	0.0231 (7)	
C10	0.2159 (2)	0.48474 (12)	0.1675 (3)	0.0212 (6)	
N2	0.90678 (17)	0.40848 (10)	0.2486 (2)	0.0228 (6)	
N3	0.91310 (17)	0.40494 (10)	0.5539 (2)	0.0221 (6)	
C11	0.8627 (2)	0.44091 (13)	0.3939 (3)	0.0227 (6)	
C12	0.9894 (2)	0.34962 (12)	0.3193 (3)	0.0244 (7)	
C13	0.9924 (2)	0.34743 (12)	0.5102 (3)	0.0247 (7)	
H2	0.70950	0.28620	0.31660	0.0350*	
H3	0.71140	0.15610	0.31560	0.0410*	
H4	0.51900	0.08860	0.17370	0.0410*	
H5	0.31170	0.15030	0.03640	0.0320*	
H9A	0.18490	0.43490	−0.09980	0.0280*	
H9B	0.32720	0.47940	−0.05150	0.0280*	
H2N	0.885 (3)	0.4263 (15)	0.114 (4)	0.061 (8)*	
H3N	0.894 (2)	0.4211 (13)	0.677 (3)	0.040 (7)*	
H11	0.80430	0.48310	0.38420	0.0270*	
H12	1.03540	0.31680	0.24770	0.0290*	
H13	1.04070	0.31250	0.59820	0.0300*	

### Atomic displacement parameters (Å^2^)

**Table:**

	*U*^11^	*U*^22^	*U*^33^	*U*^12^	*U*^13^	*U*^23^
O1	0.0257 (8)	0.0262 (9)	0.0296 (8)	−0.0052 (7)	0.0014 (6)	−0.0001 (7)
O2	0.0293 (8)	0.0287 (10)	0.0353 (9)	−0.0110 (7)	0.0026 (6)	0.0010 (7)
O3	0.0450 (10)	0.0266 (10)	0.0218 (8)	0.0109 (8)	0.0074 (7)	0.0055 (7)
O4	0.0313 (8)	0.0196 (9)	0.0195 (7)	0.0062 (7)	0.0040 (6)	0.0007 (6)
N1	0.0209 (8)	0.0159 (10)	0.0235 (9)	0.0004 (7)	0.0038 (7)	−0.0011 (7)
C1	0.0241 (10)	0.0227 (12)	0.0191 (10)	0.0015 (9)	0.0080 (8)	0.0019 (9)
C2	0.0248 (11)	0.0383 (15)	0.0259 (11)	0.0047 (11)	0.0083 (9)	0.0046 (10)
C3	0.0383 (14)	0.0365 (16)	0.0308 (13)	0.0171 (12)	0.0130 (10)	0.0083 (11)
C4	0.0523 (15)	0.0262 (14)	0.0280 (12)	0.0147 (12)	0.0168 (11)	0.0067 (11)
C5	0.0395 (13)	0.0205 (13)	0.0209 (11)	−0.0008 (10)	0.0090 (9)	−0.0030 (9)
C6	0.0270 (11)	0.0205 (12)	0.0164 (10)	0.0007 (9)	0.0091 (8)	−0.0002 (9)
C7	0.0237 (10)	0.0199 (12)	0.0174 (10)	−0.0018 (9)	0.0069 (8)	−0.0009 (9)
C8	0.0211 (10)	0.0267 (13)	0.0191 (10)	−0.0018 (9)	0.0057 (8)	0.0015 (9)
C9	0.0257 (11)	0.0199 (12)	0.0233 (11)	0.0017 (9)	0.0039 (8)	0.0016 (9)
C10	0.0217 (10)	0.0206 (12)	0.0206 (10)	−0.0034 (9)	0.0025 (8)	0.0003 (9)
N2	0.0241 (9)	0.0260 (11)	0.0190 (9)	0.0016 (8)	0.0061 (7)	0.0021 (8)
N3	0.0245 (9)	0.0233 (11)	0.0184 (9)	0.0000 (8)	0.0038 (7)	0.0003 (8)
C11	0.0223 (10)	0.0240 (12)	0.0212 (11)	0.0017 (9)	0.0025 (8)	0.0007 (9)
C12	0.0225 (10)	0.0234 (13)	0.0285 (12)	0.0052 (9)	0.0079 (8)	0.0010 (10)
C13	0.0236 (11)	0.0208 (13)	0.0289 (12)	0.0059 (9)	0.0033 (9)	0.0057 (9)

### Geometric parameters (Å, º)

**Table:**

O1—C7	1.214 (2)	C2—C3	1.391 (4)
O2—C8	1.207 (3)	C3—C4	1.389 (3)
O3—C10	1.227 (3)	C4—C5	1.403 (3)
O4—C10	1.285 (3)	C5—C6	1.380 (3)
N1—C7	1.386 (3)	C6—C7	1.488 (3)
N1—C8	1.403 (3)	C9—C10	1.524 (3)
N1—C9	1.449 (3)	C2—H2	0.9500
N2—C11	1.329 (3)	C3—H3	0.9500
N2—C12	1.375 (3)	C4—H4	0.9500
N3—C11	1.320 (3)	C5—H5	0.9500
N3—C13	1.371 (3)	C9—H9A	0.9900
N2—H2N	0.99 (3)	C9—H9B	0.9900
N3—H3N	0.97 (2)	C12—C13	1.350 (3)
C1—C8	1.485 (3)	C11—H11	0.9500
C1—C2	1.378 (3)	C12—H12	0.9500
C1—C6	1.386 (3)	C13—H13	0.9500
			
C7—N1—C8	112.13 (17)	O3—C10—O4	125.81 (19)
C7—N1—C9	124.17 (16)	O3—C10—C9	120.46 (19)
C8—N1—C9	123.69 (18)	O4—C10—C9	113.72 (17)
C11—N2—C12	108.47 (16)	C3—C2—H2	121.00
C11—N3—C13	108.44 (16)	C1—C2—H2	121.00
C12—N2—H2N	127.4 (16)	C2—C3—H3	119.00
C11—N2—H2N	124.1 (16)	C4—C3—H3	119.00
C13—N3—H3N	130.3 (13)	C3—C4—H4	120.00
C11—N3—H3N	121.2 (13)	C5—C4—H4	120.00
C2—C1—C8	130.2 (2)	C4—C5—H5	122.00
C6—C1—C8	108.52 (18)	C6—C5—H5	122.00
C2—C1—C6	121.3 (2)	C10—C9—H9A	109.00
C1—C2—C3	117.1 (2)	N1—C9—H9B	109.00
C2—C3—C4	122.1 (2)	H9A—C9—H9B	108.00
C3—C4—C5	120.4 (2)	N1—C9—H9A	109.00
C4—C5—C6	117.0 (2)	C10—C9—H9B	109.00
C1—C6—C5	122.19 (19)	N2—C11—N3	108.95 (19)
C1—C6—C7	107.83 (18)	N2—C12—C13	106.68 (18)
C5—C6—C7	129.97 (19)	N3—C13—C12	107.47 (18)
N1—C7—C6	106.14 (17)	N2—C11—H11	126.00
O1—C7—N1	124.34 (19)	N3—C11—H11	126.00
O1—C7—C6	129.5 (2)	N2—C12—H12	127.00
N1—C8—C1	105.36 (18)	C13—C12—H12	127.00
O2—C8—C1	130.18 (19)	N3—C13—H13	126.00
O2—C8—N1	124.5 (2)	C12—C13—H13	126.00
N1—C9—C10	113.57 (17)		
			
C9—N1—C7—C6	178.15 (17)	C2—C1—C6—C5	−1.2 (3)
C7—N1—C8—O2	178.4 (2)	C6—C1—C2—C3	0.3 (3)
C9—N1—C8—O2	−0.4 (3)	C8—C1—C6—C7	−1.6 (2)
C7—N1—C8—C1	−0.3 (2)	C8—C1—C6—C5	177.06 (19)
C8—N1—C7—O1	179.48 (19)	C6—C1—C8—O2	−177.4 (2)
C9—N1—C7—O1	−1.7 (3)	C6—C1—C8—N1	1.2 (2)
C8—N1—C7—C6	−0.7 (2)	C1—C2—C3—C4	0.9 (3)
C8—N1—C9—C10	−79.5 (2)	C2—C3—C4—C5	−1.3 (3)
C9—N1—C8—C1	−179.10 (17)	C3—C4—C5—C6	0.4 (3)
C7—N1—C9—C10	101.9 (2)	C4—C5—C6—C7	179.2 (2)
C12—N2—C11—N3	−0.4 (2)	C4—C5—C6—C1	0.8 (3)
C11—N2—C12—C13	0.5 (2)	C1—C6—C7—O1	−178.8 (2)
C13—N3—C11—N2	0.0 (2)	C5—C6—C7—N1	−177.1 (2)
C11—N3—C13—C12	0.3 (2)	C1—C6—C7—N1	1.4 (2)
C2—C1—C8—O2	0.7 (4)	C5—C6—C7—O1	2.7 (4)
C2—C1—C8—N1	179.3 (2)	N1—C9—C10—O4	−177.97 (17)
C2—C1—C6—C7	−179.89 (19)	N1—C9—C10—O3	2.7 (3)
C8—C1—C2—C3	−177.6 (2)	N2—C12—C13—N3	−0.5 (2)

### Hydrogen-bond geometry (Å, º)

Cg4 is the centroid of the N2/N3/C11–C13 ring.

**Table:**

*D*—H···*A*	*D*—H	H···*A*	*D*···*A*	*D*—H···*A*
N2—H2*N*···O4^i^	0.99 (3)	1.69 (3)	2.6846 (19)	178 (4)
N3—H3*N*···O3^ii^	0.97 (2)	2.54 (2)	3.087 (2)	115.4 (16)
N3—H3*N*···O4^ii^	0.97 (2)	1.71 (2)	2.680 (2)	175 (2)
C3—H3···O4^iii^	0.95	2.45	3.321 (3)	153
C5—H5···O3^iv^	0.95	2.48	3.266 (3)	141
C9—H9*A*···O1	0.99	2.55	2.891 (3)	100
C9—H9*B*···O2^i^	0.99	2.41	3.397 (3)	172
C11—H11···O3^ii^	0.95	2.40	2.987 (3)	120
C13—H13···O1^v^	0.95	2.54	3.352 (2)	143
C2—H2···*Cg*4	0.95	2.87	3.805 (2)	166

Symmetry codes: (i) −*x*+1, −*y*+1, −*z*; (ii) −*x*+1, −*y*+1, −*z*+1; (iii) −*x*+1, *y*−1/2, −*z*+1/2; (iv) *x*, −*y*+1/2, *z*−1/2; (v) *x*+1, *y*, *z*+1.
