# Supplementary figures and images for: Crystal structure of 1H-imidazol-3-ium 2-(1,3-dioxoisoindolin-2-yl)acetate
Source: Acta Crystallogr Sect E Struct Rep Online. 2014 Aug 6;70(Pt 9):o979–80. doi: 10.1107/S1600536814017619 (PMC4186205; doi:10.1107/S1600536814017619)

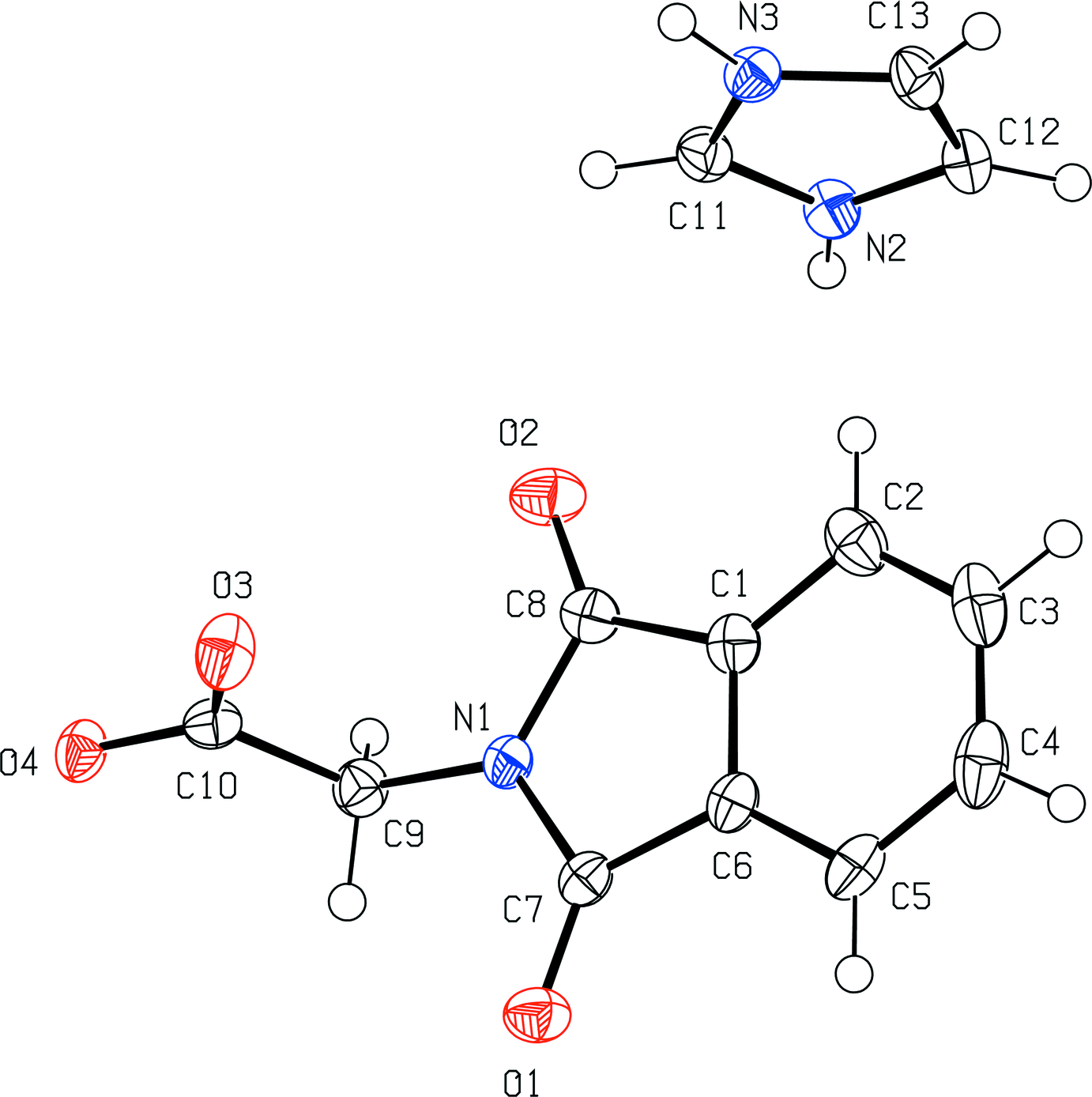

Supplement: Supplementary file 4 [file e-70-0o979-fig1.tif]

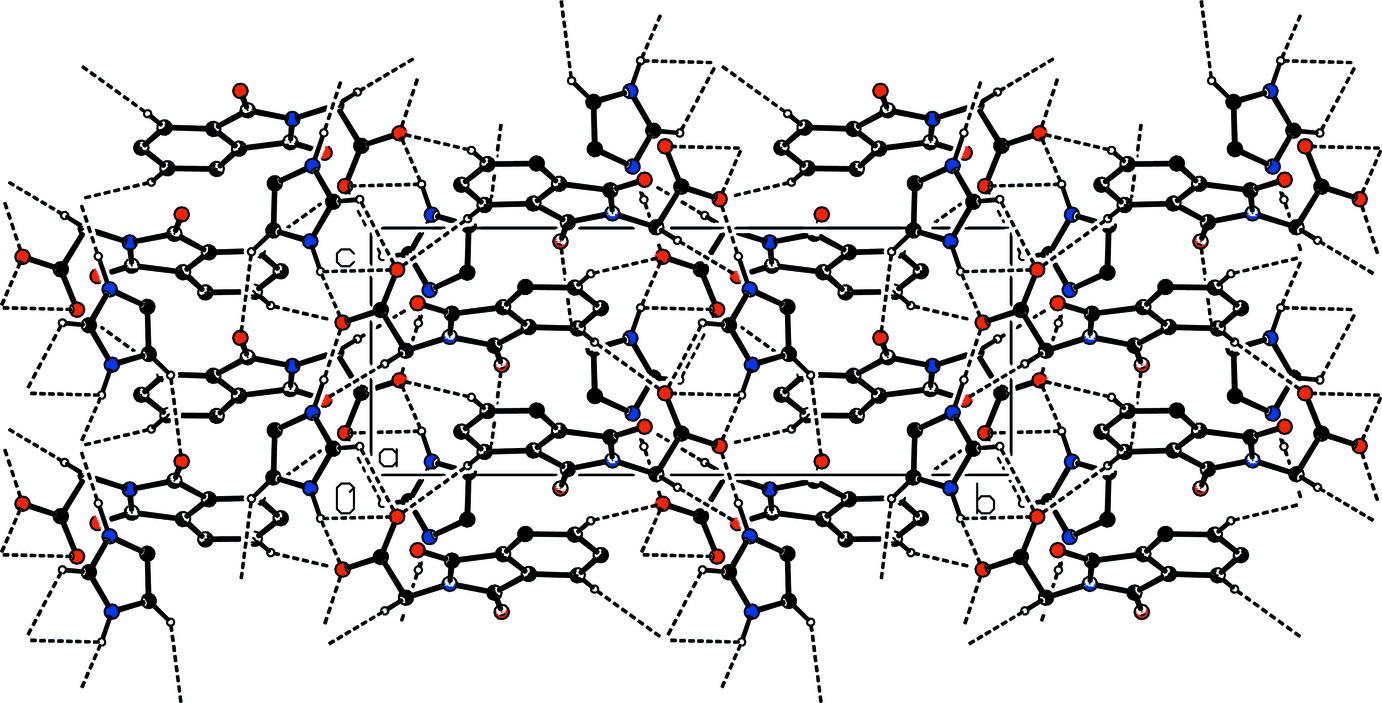

Supplement: Supplementary file 5 [file e-70-0o979-fig2.tif]
